# Towards Stratified Medicine in Plasma Cell Myeloma

**DOI:** 10.3390/ijms17101760

**Published:** 2016-10-21

**Authors:** Philip Egan, Stephen Drain, Caroline Conway, Anthony J. Bjourson, H. Denis Alexander

**Affiliations:** Northern Ireland Centre for Stratified Medicine, Biomedical Sciences Research Institute, Ulster University, C-TRIC Building, Altnagelvin Area Hospital, Glenshane Road, Derry/Londonderry BT47 6SB, Northern Ireland; egan-p2@email.ulster.ac.uk (P.E.); s.drain@ulster.ac.uk (S.D.); c.conway@ulster.ac.uk (C.C.); aj.bjourson@ulster.ac.uk (A.J.B.)

**Keywords:** plasma cell myeloma, multiple myeloma, plasma cell dyscrasias, personalised medicine, flow cytometry, Next Generation Sequencing, proteasome inhibitors, immunomodulatory drugs, microRNAs

## Abstract

Plasma cell myeloma is a clinically heterogeneous malignancy accounting for approximately one to 2% of newly diagnosed cases of cancer worldwide. Treatment options, in addition to long-established cytotoxic drugs, include autologous stem cell transplant, immune modulators, proteasome inhibitors and monoclonal antibodies, plus further targeted therapies currently in clinical trials. Whilst treatment decisions are mostly based on a patient’s age, fitness, including the presence of co-morbidities, and tumour burden, significant scope exists for better risk stratification, sub-classification of disease, and predictors of response to specific therapies. Clinical staging, recurring acquired cytogenetic aberrations, and serum biomarkers such as β-2 microglobulin, and free light chains are in widespread use but often fail to predict the disease progression or inform treatment decision making. Recent scientific advances have provided considerable insight into the biology of myeloma. For example, gene expression profiling is already making a contribution to enhanced understanding of the biology of the disease whilst Next Generation Sequencing has revealed great genomic complexity and heterogeneity. Pathways involved in the oncogenesis, proliferation of the tumour and its resistance to apoptosis are being unravelled. Furthermore, knowledge of the tumour cell surface and its interactions with bystander cells and the bone marrow stroma enhance this understanding and provide novel targets for cell and antibody-based therapies. This review will discuss the development in understanding of the biology of the tumour cell and its environment in the bone marrow, the implementation of new therapeutic options contributing to significantly improved outcomes, and the progression towards more personalised medicine in this disorder.

## 1. Introduction

Plasma cell myeloma (PCM), or multiple myeloma (MM), is an incurable haematological malignancy of end stage B lineage cells, or plasma cells [1]. Normal plasma cells form a vital arm of the adaptive immune system, each secreting antibody of differing specificity that circulates throughout the body, whereas myeloma plasma cells in a patient all secrete antibody having a single, or monoclonal, specificity. On serum electrophoresis, this is manifested by the presence of a monoclonal, or paraprotein, band, the so-called M band.

PCM has an incidence of approximately 65 new cases per million of the population per annum and constitutes around one to 2% of all cancers. It is the third most common B cell malignancy after diffuse large B cell lymphoma (DLBCL), closely following chronic lymphocytic leukaemia (CLL) in frequency, and has a male:female ratio of around 1.3:1. There has been a significant rise in the incidence of PCM since the 1960s. It has a median age at presentation of 70 years, and 43% of patients are 75 years old and above [2,3]. Thus, its increasing frequency may be explained by evolving demographics, underpinned by an improving diagnostic pick-up rate. Overall survivals have improved during the past two decades from a median of 3–4 years to 5–7 years, although upwards of 25% of those diagnosed survive for less than 24 months. 50%–70% of patients live for five years or longer, depending on their response to, and tolerance of, drugs used in their treatment [4]. This improvement in outcomes is attributable to innovative treatments including autologous stem cell transplantation (SCT) and the widespread use of proteasome inhibitors and immunomodulatory drugs. Major obstacles to improved outcomes are the disease’s heterogeneity, drug resistance and the immunosuppressive nature of the tumour in its bone marrow microenvironment [5].

Monoclonal gammopathy of undetermined significance (MGUS), smouldering multiple myeloma (SMM), and plasma cell leukaemia (PCL), along with PCM, are classified as plasma cell dyscrasias (PCD) [6]. MGUS is present in three to 4% of the population over the age of 50 years and the incidence increases with age. Its prevalence is influenced by various factors in addition to age, including race, gender, family history, immunosuppression and exposure to pesticides [7,8]. The World Health Organisation (WHO) 2016 classification separates MGUS into IgM and IgG/A subtypes and the former is nearly always a precursor of lymphoplasmacytic lymphoma, including Waldenström macroglobulinemia, rather than PCM [1]. The existence of IgM myeloma is still questioned by some authorities and it is certainly uncommon, constituting <1% of all cases. Approximately 1% of cases of MGUS progress to PCM per annum. SMM constitutes approximately 14% of all cases of myeloma and carries a 10% risk per annum of progression to PCM. As with PCM, it is a heterogeneous condition [7].

There are marked differences in the incidence of PCM across ethnic groups. For example, in North America it is twice as common in people of African-American origin as in Caucasians but the former are under-represented in clinical trials [2,9]. MGUS follows this pattern with, for example, prevalence in individuals >50 years old of 5.9%–8.4% in black people, 3.0%–3.6% in white, and even lower in the Japanese population [8]. More research is required into biological and socioeconomic differences between ethnic subgroups in PCM [9].

In this review, we summarise current knowledge of the biology of PCM and its precursor conditions MGUS and SMM, and provide an in-depth account of biomarkers contributing to risk stratification in these disorders. We review therapeutic options, including novel therapies undergoing clinical trials and discuss progress towards stratified, or personalised, medicine for those diagnosed with these disorders. Finally, we outline what we perceive to be the principal challenges remaining before significant progress towards a cure is realised.

## 2. Tumour Biology

B cells are derived from haematopoietic stem cells in the bone marrow (BM) and during B cell ontogeny there are at least three seminal events that may provide windows of opportunity for mistakes to occur at a molecular level, which, in most instances, result in the death of the cell. However, some result in a proliferation or survival advantage, the latter through resistance to death by apoptosis. These events are: (1) Immunoglobulin (*Ig*) gene rearrangement, which occurs in early B lineage cells in the bone marrow. In order to generate antibody diversity, germline DNA is rearranged. This involves variable (V), diversity (D) and joining (J) segments of the *Ig* gene, which are rearranged in an orderly fashion to generate the very wide potential Ig repertoire, thought to be in the region of 10^12^ B lineage cells and their progeny, each producing an Ig with an exquisitely specific antigen recognition site; (2) somatic hypermutation, which occurs following antigenic presentation, usually in germinal centres of the lymph nodes. This leads to a fine adjustment of the antibody binding site, enabling a better “fit” between antigen and Ig, somewhat analogous to tailored modification of an “off the peg” garment; (3) class switching, which takes account of the antigenic stimulation of the B cell and the most appropriate Ig class of antibody to eradicate that pathogen. Thus, IgM production, which is the Ig produced before antigen presentation, is switched to IgG, IgA, or IgE, and the progeny cells then become memory B cells, able to mount an immediate response if presented again with the same antigen, or plasma cells, which are the end stage B lineage cells, able to produce copious amounts of antibody.

Mistakes during any of these three seminal stages are thought to be critical events promoting oncogenesis in B lineage cells. As less than 1% of PCM involve IgM producing cells, successful class switching has occurred in the remainder and this would seem to suggest that the mutagenic “hit”, which has resulted in the growth of a monoclonal population of plasma cells has occurred at a later, rather than earlier, stage of B cell ontogeny, and the somatic hypermutation and Ig class switching stages are plausible candidates. Earlier reports of a “stem cell like” early B lineage cell being found in BM aspirate from PCM patients have not been substantiated, nor is there universal acceptance of reports that Ig light chain restricted B lymphocytes are present in peripheral blood (PB) from PCM patients. Methodological considerations are especially important because of the presence of large amounts of paraprotein in the PB of patients, which, if steps are not taken to remove adhered Ig from the lymphoid cells before testing, may be wrongly interpreted as evidence of monoclonal Ig production by those cells.

Despite its noted clonal origin, PCM is a disease that manifests considerable molecular heterogeneity and genomic instability, the latter being the highest of any haematological malignancy. Commonly detected acquired chromosomal aberrations in PCM patients include translocations of the *IGH* gene (chromosome 14) that constitutively activate other oncogenic pathways including those involving products coded for by genes *CCND1*, *CCND3*, *MAF*, *MAFB* and *FGFR3/MMSET* [10,11,12]. The manifestation of these aberrations is also highly variable; 50% per cent of PCM tumours are typically hyperdiploid and demonstrate trisomies of certain chromosomes whilst the other half are often hypodiploid and here monosomy, particularly of chromosomes 1, 13 and 11, is often seen [11]. Clinical detection of these cytogenetic aberrations has allowed for the first substantial risk based stratification of PCM patients in the history of this disease [13,14]. However, there have been very few links made between this initial risk stratification and the treatment options available for PCM [12]. More recent evidence has also shown that different combinations of these cytogenetic aberrations can have different impacts on risk stratification. For example, the presence of trisomies has been shown to mitigate the adverse prognostic impact of high-risk cytogenetics such as t(4;14), t(14;16) and t(14;20) [11].

Interphase fluorescence in-situ hybridisation (iFISH) is the current gold standard method used to identify acquired chromosomal aberrations but it is a targeted approach. Integrative genomics, including new approaches such as gene expression profiling (GEP) and whole genome sequencing (WGS) will therefore be pivotal, going forward, to screen for all possible chromosomal aberrations to identify those present on a case by case basis, potentially discovering new aberrations in the process that are of relevance in unravelling the complex biology of the tumour cells.

Deregulation of non-coding RNAs has also been shown to be an important factor in the biology of PCD, adding an additional level of complexity to the molecular understanding and risk stratification, and providing opportunities for a novel therapeutic approach [15,16,17,18,19,20]. MicroRNAs (miRNA) and long non-coding RNAs (lncRNA) have been shown to regulate the expression and function of other genes, normally via direct interference during translation [21,22]. Recently, in PCM, overexpression of the *MALAT1* transcript has been shown, which in turn leads to over activation of the *LTBP3* gene and changes to cell cycle regulation and messenger RNA (mRNA) maturation processes [22,23]. These studies also demonstrated that a further 21 lncRNAs became progressively down-regulated in samples representing the more aggressive stages of PCM and that a transcriptional fingerprint could be identified in hyperdiploid PCM patients which could further enhance current cytogenetic based risk stratification strategies [16,22,23,24].

miRNA expression patterns (miREPs) have demonstrated significant differences when the miREPs of healthy donor plasma cells (PC) are compared with those from PCM tumour PC, and also when comparing the different clinical stages of PCD (MGUS, SMM, PCM) [16,17,25]. Furthermore, cluster analyses have shown that the miRNA regulatory network in PCM is complex and involves interactions with several newly discovered oncological pathways such as the Hippo and acute myeloid leukaemia (AML) associated pathways [16]. It should be noted that in most of these studies the prognostic, as defined by stage specific, differences in miREPs were less significant than the diagnostic (i.e., normal vs. PCD), suggesting a primary role in the establishment of the initial PC tumour, rather than its progression [17,25]. However, it has also been shown that miREPs can be correlated with specific cytogenetic aberrations, with the strongest correlations seen between certain miRNAs such as miRNA-99b and high-risk translocations involving the *IGH* gene, such as t(4;14). This may suggest a separate role in already progressed PCM and is further evidence that the relationship between cytogenetic aberrations and miREPs is extremely complex [17].

Other integrative genomics approaches have demonstrated that miRNA expression, specifically miR-335, miR-342 and miR-561, can be correlated with deregulated host genes (*MEST*, *EVL* and *GULP1*) and that these genes may play a role in the bone marrow microenvironment and the homing interactions of the plasma cell within this environment [15,21]. Again, it should be noted that different combinations of these genetic lesions can have considerable differences in terms of individual patient prognosis and indeed more routine use of integrative genomics approaches is advocated to ensure that all potential aberrations and the combinations of these can be comprehensively screened for in PCM patients [16,22].

Whole genome and exome sequencing has enhanced understanding in this field. For example, in a study of over 200 PCM tumours, Lohr et al. found each contained 3–7 subclones [26]. Clonal heterogeneity has also been identified using serum electrophoresis where more than one paraprotein species is sometimes detected in the same patient, cytogenetics where acquired aberrations are frequently found in only a proportion of the myeloma cells, and through differing treatment responses from BM and extramedullary lesions [27].

The high genomic instability of myeloma compared to other haematological malignancies results in tumours likely to contain genetically distinct subclones, potentially with different levels of drug resistance. One study which followed 24 patients from diagnosis, through treatment and relapse observed three patterns in clonal evolution: no change in the subclones between diagnosis and relapse; the major clone at diagnosis acquired further genetic lesions and caused relapse; a minor clone at diagnosis became the dominant clone, presumably through drug resistance, and caused relapse [28].

Furthermore, the often complex nature of changes in gene copy number in sequential samples suggests an evolution of tumour subclones [27]. Sequential molecular analyses through the course of the disease have allowed models of Darwinian clonal evolution to be postulated, where subclones can become dominant over time or following treatment. One group proposed a clonal evolution model with four alternatives: (1) no change; (2) a change in the proportions of each subclone due to a different treatment response; (3) linear evolution producing a new clone at relapse; and (4) branching evolution producing two new clones at relapse [27]. An example of clonal evolution in progress was described by Raab et al, where a patient with refractory PCM was treated with the B-Raf inhibitor vemurafenib, leading to the emergence of a resistant clone. Thereafter, the patient was treated with either or both B-Raf and proteasome inhibitors in response to clonal evolution in order to target spatially separated, B-Raf inhibitor sensitive and resistant clones [29].

Drugs are usually used in combinations and identifying the most effective for each subtype and stage of disease is currently the subject of several clinical trials. A cocktail of anti-cancer agents might have greater efficacy against a tumour containing subclones; indeed, the level of tumour heterogeneity might in future be used to determine the number of different agents in a treatment combination [30,31]. However, such an approach is often accompanied by increased toxicity and may be out-ruled because of this.

## 3. Investigation of Suspected Plasma Cell Dyscrasias

Suspected PCD are investigated at presentation by a multifaceted approach for both diagnostic and prognostic purposes. This includes: detection of paraprotein, or M band in serum or urine by immunofixation electrophoresis and/or nephelometry, examination of BM aspirate and BM trephine biopsy using morphology, and flow cytometry or immunohistochemistry (IHC), respectively, iFISH to identify recurring acquired cytogenetic aberrations, imaging techniques to identify the presence of bone lesions detected by skeletal survey and the presence of one or more of hypercalcaemia, renal insufficiency, anaemia, bone lesion CRAB criteria (Table 1) [32].

PCL is defined as having 2 × 10^9^ plasma cells/L PB or, where there are <10 × 10^9^ leucocytes/L PB, 20% of leucocytes are plasma cells. It can be a primary malignancy (de novo presentation), or appear as a late, usually terminal stage of myeloma [33]. Newer methods are augmenting the above diagnostic and prognostic methods and contributing to understanding of tumour biology in PCM, including causes of relapse and drug resistance. For example, multiplex ligation-dependent probe amplification (MLPA) is increasingly employed, in addition to the well-established iFISH technique, to detect chromosomal aberrations. Research studies are employing Next Generation Sequencing (NGS) methods, while GEP have shown prognostic power. However, these newer methods are not yet established in most clinical centres and await implementation in patient management.

There is an ever-expanding range of laboratory-based techniques being used to explore the biology of the malignant cells and their microenvironment [14]. Knowledge of the previous treatments received is relevant in terms of toxicities suffered by the patient and, together with response to treatment, provide clues as to tumour drug resistance [34]. With recent advances in immunotherapies, focus has turned to the tumour’s interaction with its microenvironment and with the cells of the immune system in particular.

Molecular methods can be used for detection of minimal residual disease (MRD), detection of clonal heterogeneity and shifts in the clonal make-up of the tumour, gene expression profiling and subsequent identification of drug targets. For highly sensitive detection of myeloma cells, allele-specific oligonucleotide real-time quantitative PCR (ASO PCR) is a viable alternative to flow cytometry [35], while NGS is a powerful tool for elucidating the tumour’s clonal hierarchy, and gene expression arrays linked to outcome data allow identification of groups of key genes with strong prognostic value.

## 4. Risk Stratification

The aim of risk stratification is to obtain the most meaningful assessment of a patient’s likelihood of disease progression, potential resistance to specific therapies and to inform therapeutic management decisions [36,37]. It can also contribute to reducing overtreatment, which, in addition to minimising patient discomfort, lowers risk of genotoxicity and consequent mutation-induced drug resistance [38]. Patients themselves may benefit from a more informed understanding of their illness, treatment options where they exist, and likely prognosis [38]. Furthermore, investigations contributing to risk stratification allow data on populations in different clinical trials to be compared more effectively by having better-defined subsets of patients [32,37,38].

Without strong evidence linking risk group and treatment regimen with outcome, the application of risk stratification to treatment decisions may be inappropriate [32]. Several prognostic markers have been identified in PCM but none of the current risk stratification systems is adequately robust to fully inform treatment decisions, largely due to the heterogeneity of the disease [37].

PCM patients are assessed at diagnosis using the International Staging System (ISS) and assigned to stage 1, 2 or 3 based on the concentration of β2-microglobulin and albumin in their serum [39]. β2-microglobulin levels increase with increasing stage while albumin levels are reduced [39]. The ISS stages are prognostically significant and form the basis for all risk stratification systems [14,38]. It is informative of patient fitness and tumour burden [32,40]. ISS has been well validated, and is applicable in any part of the world [37].

The International Myeloma Working Group (IMWG) defined a high-risk group as having a median overall survival (OS) <2 years even with the latest treatment, and a low risk group as having a median OS >10 years [37]. The ISS was revised in 2015 by combining it with iFISH data to add a tumour biology component and serum lactate dehydrogenase (LDH) level as a further measure of tumour burden [41], although this information may not always be routinely available (Table 2) [42].

In an alternative risk stratification system, Usmani et al. devised high- and ultra-high-risk categories with <3 year and <2 year median OS, respectively [42]. The ultra-high-risk group was defined using high-risk chromosomal aberrations, LDH, ISS, treatment response, MRD and presence of circulating plasma cells in PB, although an individual might only need to show one of these characteristics. However, this is still a heterogeneous group [42].

### 4.1. Types of Risk Stratification Parameters

Risk stratification investigations often overlap with the diagnostic procedures outlined above and increasingly form an integrated approach with these in the initial assessment of a newly presenting patient. The clinical and laboratory data that can inform treatment decisions fit into four main categories: patient factors, tumour burden, cytogenetics and molecular genetics, in particular GEPs [14].

#### 4.1.1. Patient Factors

Patient factors include age, fitness, related organ or tissue impairment and other co-morbidities [14]. Age is the most important of these although this may be as a surrogate for other factors such as frailty, in which case biological age would be more appropriate [9,37]. Patients over 80 years old have an inferior performance status and more advanced disease, both of which are independent markers of poor OS [33]. Co-morbidity, especially renal insufficiency, is an important marker of high-risk [9,42] and the ISS incorporates a measure of renal function. Renal insufficiency has been shown to occur in about a third of patients but the appropriate treatment can reverse this and effectively overcome it as a negative indicator [33].

Although the median age at diagnosis of PCM is approximately 70 years, the full effect of age is difficult to ascertain since most interventionist clinical trials tend to enrol younger patients who are more likely to be 60–65 years old, and these may have been diagnosed several years earlier [9].

#### 4.1.2. Tumour Burden

This can be measured by histological examination of BM trephine biopsy specimens, imaging, flow cytometry and blood biochemistry, with the last of these used in the ISS. Imaging may use radiography, magnetic resonance imaging (MRI), computed tomography (CT) and positron emission tomography (PET) [43]. MRI and PET are particularly effective in identifying extramedullary disease. Conventional radiography has been the standard technique, mainly due to availability and relatively low cost. However, for example, fluorodeoxyglucose positron emission tomography (FDG-PET) provides additional data of prognostic value with the number of focal skeletal lesions, the presence of extramedullary lesions, and standardised uptake of FDG, all shown to be correlated with OS [33]. Extramedullary disease has been reported to reduce five year OS from 59% to 31%, is associated with fractures and may steer treatment towards cytotoxic drugs instead of novel agents [9,33].

Serum LDH level gives a measure of tumour metabolism from which one can infer tumour burden and is used in the revised ISS (R-ISS). Similarly, proliferation activity can be measured by plasma cell labelling index (PCLI) [37]. PCLI is a measure of the percentage plasma cells in synthesis phase of life cycle, i.e., proliferation, and is prognostically relevant, with higher values associated with significantly shorter OS.

Measurement of paraprotein, whether whole Ig or a light chain subunit, is of considerable prognostic relevance. Identification of the Ig subtype is necessary for a definitive diagnosis and has further significance. For example, an IgA MGUS or SMM has a four times higher risk of progressing to PCM than other subtypes [44], whilst IgD PCM has been associated with poorer outcomes. Suppression of Ig subtypes other than the subtype of the plasma cell clone is also associated with an increased risk of progression from precursor to PCM [44], and with clinical status and progression-free survival (PFS) in PCM [45]. Paraprotein identification in MGUS allows categorisation into IgM, non-IgM and light chain MGUS, which are clinically distinct conditions [7].

As there is evidence that high- and low-risk subtypes exist within MGUS and SMM, this raises the possibility of extending risk stratification to MGUS and SMM using paraprotein measurement alongside cytogenetics and molecular genetics and multiparameter flow cytometry [46]. Monitoring MGUS patients before they develop PCM has been shown to be beneficial after a study of approximately 17,000 patients in the US between 1994 and 2007 demonstrated that those given further check-ups had significantly better OS [47]. Trials using myeloma-targeting monoclonal antibody (mAb) monotherapy in SMM are in progress and this may in turn lead to MGUS being treated as the antibody therapy may represent a way of reducing risk of transformation to PCM with minimal treatment-induced toxicity [48]. Treatment of high-risk SMM has been shown to be effective [7].

Further risk factors include serum free light chains (FLC), heavy to light chain ratio [32] and κ to λ free light chain ratio (rFLC) [49]. rFLC may be useful in oligosecretory PCM, which may represent >10% of cases, for monitoring and to allow their inclusion in clinical trials [49]. Serum B cell maturation antigen (BCMA) is found in MM at higher levels than in MGUS and healthy controls. Levels are higher in progressive than in stable disease and there is an inverse correlation with OS [50]

#### 4.1.3. Cytogenetics

Interphase FISH (iFISH) is the current gold standard for detection and identification of acquired cytogenetic abnormalities in BM aspirates. When tumour burden is low plasma cells may be enriched by selection using anti CD138 mAb conjugated to microbeads [33].

Many of the aberrations are chromosomal translocations involving *IGH* genes on chromosome 14. These are thought to be early events in B cell ontogeny post BM stage, possibly due to error(s) occurring following antigen presentation in germinal centres of lymph nodes, when *IGHV* somatic hypermutation and class switching occur [51]. The aberrations most associated with a poorer prognosis are t(4;14), del(17p13), del(13q), and del(1p), especially when more than one of these is present [33,37,52]. Evidence is weaker regarding possible prognostic association with t(14;16), t(11;14) and add(1q21), all of which are also found in PCM, although the IMWG R-ISS includes t(14;16) as a poor prognosis aberration [37,52]. Of considerable importance in informing therapeutic decision making is evidence that the proteasome inhibitor bortezomib has been shown to overcome OS and PFS disadvantages in patients with del(13q) and at least improve outcomes in patients with t(4;14) or del(17p) (11).

A powerful example of the prognostic significance of high-risk acquired chromosomal aberrations, when combined with GEP, is illustrated in the Mayo Clinic score. In this model, either a GEP associated with a shorter OS or any of three high-risk chromosomal aberrations, namely del(17p), t(14;16) or t(14;20), define a high-risk group of around 20% of newly diagnosed patients, with a median survival of three years. A further 20% lack any high-risk markers but are defined as intermediate risk by either hypodiploidy, PCLI >3%, t(4;14), or del(13), with a median OS of 4–5 years. The remaining 60% of new cases are standard-risk, with a median OS of 8–10 years even though chromosomal aberrations t(11;14) or t(6;14) may be present (Table 3) [14].

#### 4.1.4. Gene Expression Profiles

Several groups have proposed GEPs for high-risk myeloma. For example, the GEP70 was devised using 532 newly diagnosed myeloma patients. Seventy genes were found to be linked independently to lower OS, duration of complete response (CR) and event-free survival (EFS) [53]. This GEP was then used to identify high-risk subgroups within MGUS, SMM and newly-diagnosed PCM patients [53]. In addition, GEP70 seemed to confirm the cytogenetic observations relating to loss of 1p and gain of 1q as 30% high-risk genes were found to be on this chromosome and genes on 1p were downregulated while those on 1q were upregulated [53].

The Intergroupe Francophone du Myélome collaboration identified 15 genes related to control of the cell cycle with the most profound negative effect on OS from 182 newly diagnosed PCM patients [33]. Kuiper et al. analysed several data-sets encompassing 4750 patients and found the strongest predictor of risk was the EMC92 gene panel used in concert with ISS with which they could risk stratify into four categories with median OS of 24, 47, 61 and >96 months [54]. Work is on-going to produce a standardised GEP to allow comparison of different study populations, although its cost may prove a barrier to widespread use [14,33,37].

Putative drug resistant cells have shown gene expression to be both generally dysregulated and specifically altered involving, for instance, genes with a role in the cell’s processing of protein seen in relapsed proteasome inhibitor treated patients [28,55]. In addition, expression of multi-drug resistance (*MDR*) genes such as ATP binding cassette (*ABC*) genes can change after therapy [56]. Detailed knowledge of such changes in gene expression may lead to treatment choices for relapsed patients being directed by genetic signatures of drug resistance [9].

A specific gene expression pathway or product is a potential drug target if overexpression or mutation is associated with an unfavourable outcome, especially if these occur in tumour progenitor cells so that they are present in most or preferably all tumour cells [57]. Knowledge that the gene plays an important role in driving malignancy, such as encoding a key signalling protein such as MAPK or c-Jun, increases this potential [58]. However, the presence of another mutation in the same pathway could render targeting only one of them ineffective [26]. Furthermore, specific targeting creates a selection pressure and with it the potential for the emergence of a resistant clone; for example, resistance to B-Raf inhibitors in extramedullary disease after selection of neuroblastoma RAS viral oncogene homolog (*NRAS*) mutated subclones [29].

Very recent evidence suggests that overproduction in PCM of a regulator of G-protein signalling coded for by the *RGS1* gene and demonstrated by an immunohistochemical technique on BM trephine biopsy samples, correlated with shorter OS [59]. If confirmed, this would be a readily accessible procedure for incorporation in routine investigations in PCD.

Finally, GEP and NGS identified genes whose malfunction is known to contribute to other cancers, while inactivation of others conferred a favourable outcome. The genes identified include *BRAF*, *KRAS*, *NRAS*, *MAPK*, *MYC*, *FAM46C*, *SP140*, *LTB*, *EGR1*, *IRF4*, *MAX*, *HIST1H1E*, *RB1*, *EGR1*, *TP53*, *TRAF3*, *DIS3*, *CYLD*, *FGFR3*, and *NFκB* [57,60,61].

## 5. Treatment

Myeloma treatment is divided into several stages: induction, consolidation and maintenance [57]. Induction is designed to de-bulk the tumour; consolidation to remove all remaining tumour cells including, hopefully, subclonal populations; and maintenance therapy is designed to alter the selection pressure on any remaining tumour cells so that less aggressively malignant cells are favoured.

Induction usually involves the use of combination therapy including a cytotoxic drug. In addition to significant toxicity these drugs have the potential to cause mutations and hence can drive clonal evolution, which may produce drug-resistant subclones, with some evidence, for example, that immune modulators may cause further mutations [57].

Upon diagnosis of PCM, options for induction therapy include cytotoxic drugs, SCT, immune modulators, proteasome inhibitors and corticosteroids, either as monotherapy or in combination. A patient’s age and co-morbidities are taken into account when making decisions on an initial course of treatment. Renal function is of particular importance and affects choice of drug and dosage [32]. SCT is used mainly in younger, fitter patients, with those over 75 years old, and/or with co-morbidities, deemed in many centres to be ineligible for transplant, although selection criteria vary from centre to centre. For maintenance and salvage therapy, previous treatment response, drug resistance and toxicity are important factors [32]. There are few routinely available predictive biomarkers which provide a robust assessment of likely response to specific treatment but as more are identified these will be increasingly important in informing treatment strategies [37].

The most significant advances in drug therapies during the past two decades have been the introduction of proteasome inhibitors and the immunomodulatory drugs. Proteasome inhibitors such as bortezomib and carfilzomib interrupt the ubiquitin proteasome system (UPS) and thus trigger apoptosis via an accumulation of proteins that may include pro-apoptotic factors and reactive oxygen species (ROS) that myeloma cells, heavily reliant on protein homeostasis, cannot tolerate [62,63]. Normally, build-up of unfolded protein in the endoplasmic reticulum triggers the unfolded protein response (UPR) via sensor proteins PERK, ATF6 and IRE1. It is thought UPR activation can lead to autophagy and upregulation of the UPS, averting crisis by reducing the amount of accumulated protein.

The first in class proteasome inhibitor bortezomib has been shown to be effective in consolidation therapy, increasing depth of treatment response, and in maintenance therapy, allowing longer term disease control [64]. Its disruption of the proteasome also helps reverse the bone loss that is a frequent feature of myeloma by preventing the proteins that stimulate bone formation from being degraded [65]. The second generation proteasome inhibitor carfilzomib has shown efficacy in relapsed or refractory PCM patients and is now being trialled in newly-diagnosed PCM and high-risk SMM patients [66].

The overall effectiveness of proteasome inhibitors is limited by the resistance the disease eventually develops. Resistance has been linked to a reduced UPR, leading to speculation that mechanisms such as autophagy, deubiquitylation and heat shock response allow the cell to overcome this stress [67]. This would suggest disease resistance across a broad range of proteasome inhibitors whereas resistance gained via changes in proteasome subunit structure, or expression, might be overcome by a different proteasome inhibitor [68].

Immunomodulators (IMiDs) target the tumour microenvironment, reducing growth signals the tumour benefits from and making immune evasion less likely by priming NK cells and inhibiting regulatory T cells [63]. Thalidomide, lenalidomide and pomalidomide, first to third generation drugs respectively, are used in maintenance therapy, having the advantage of being taken orally although toxicity can be a problem [64]. They have also been shown to be effective in relapsed or refractory patients [69].

### 5.1. Treatment Response

The standard response categories set out by the IMWG range from stringent complete response (sCR) to stable disease (SD) as defined by paraprotein levels in blood and urine, the percentage plasma cells in a bone marrow trephine biopsy, and the absence of cytoma(s). The sCR category was added due to the availability of sensitive biochemical techniques and is defined by an absence of detectable clonal serum FLC [70,71,72]. The categories are: stringent Complete Response (sCR); Complete Response (CR); Very Good Partial Response (VGPR); Partial Response (PR); Minimal Response (MiR); Stable Disease (SD).

Depth of response is related to OS [55]. New treatments have higher efficacy and therefore require more sensitive assessment of responses [71]. Further response categories added in 2016 are defined by the absence of minimal residual disease (MRD), that is, the absence of clonal plasma cells detectable by multiparameter flow cytometry or molecular techniques [73]. Hence the IMWG MRD criteria define sustained MRD-negative, flow MRD-negative, sequencing MRD-negative and imaging-positive MRD-negative categories [73]. Imaging is important for staging at presentation, but can also be useful for assessment of treatment response, as acknowledged in these new categories. MRI is the most sensitive imaging method for spinal lesions whereas PET-CT is the optimum technique for detection of extramedullary disease [71].

Currently, most hospital investigations do not routinely look for MRD by immunophenotypic or molecular methods but by doing so the MRD categories of treatment response could prove informative. In one study, multiparameter flow cytometry was able to detect MRD in 14.5% of patients who had shown a CR [74]. Paraprotein can have a long half-life so there may be a considerable time lag after tumour has been depleted before sFLC before undetectable [72]. In addition, measurement of response to mAb treatments may be complicated by similarity between the treatment antibody and the paraprotein. This could lead to confusing electrophoresis data were it not for sample pre-treatment to specifically alter the way the treatment antibody runs on the electrophoresis so it can be distinguished from paraprotein [48].

### 5.2. Link between Response Depth/Duration and Outcome

Patients achieving CR gain a longer OS [33]. Patients whose disease proves refractory to immunomodulators and proteasome inhibitors, the so-called novel therapies, have a median OS of around nine months and new treatment strategies are therefore needed urgently for this cohort of patients [48]. Duration of response is known to decrease with successive rounds of treatment [9]. On the other hand, re-treatment with the same agent is feasible if there was at least a PR the last time it was used [9]. However, drug resistance occurs eventually in almost all patients.

MRD is an independent prognostic factor that can also predict a poorly sustained CR [33]. The inclusion of the MRD response categories has enabled patients previously categorised as CR to be split into groups with significantly different OS [33]. Obtaining CR may not be as important in those with low-risk disease identified by GEP [37].

The potential usefulness of response level in risk stratification has been highlighted by studies that have shown that the benefit from a second SCT is dependent upon previously achieved response level, with lower responses benefiting [33,42]. However, as understanding of tumour biology improves, the underlying factors contributing to CR or lack thereof will become clearer and these are likely to supplement, and possibly replace, treatment response as prognostic factors [33]. Interestingly, high-risk patients may obtain a similar response to induction therapy using novel treatments as those with low-risk disease but have a shorter PFS and OS [57].

Some cases of PCM share characteristics with MGUS in that they are indolent tumours. It has been suggested that a deep response to treatment in these patients may even reduce OS through eradicating the more indolent clone and leaving a window of opportunity for expansion of a drug resistant clone [57]. Although treatment of high-risk SMM is also associated with a similar concern, a recent clinical trial in SMM using a combination of lenalidomide and dexamethasone has demonstrated encouraging efficacy [57]. However, in many centres, SMM patients are still managed by a watch and wait approach, with careful monitoring of clinical, biomarker and imaging indictors of disease progression before a decision to commence treatment is reached.

### 5.3. Overcoming Prognostic Disadvantage

Some new treatments can overcome the prognostic disadvantage of particular cytogenetic aberrations and produce the same outcome as for standard risk patients [34]. As stated previously, the proteasome inhibitor bortezomib has been shown to overcome OS and PFS disadvantages in patients with del(13) and at least improve outcome in patients with t(4;14) or del(17p) [34]. Thus del(13) is no longer seen as a high-risk chromosomal aberration if treatment with bortezomib is used [33]. The immune modulator pomalidomide has shown similar effects in relapsed/refractory PCM with del(17p) [42,75]. This has led to new risk stratification models such as the Mayo Stratification of Myeloma and Risk-Adapted Therapy (mSMART) guidelines where an intermediate risk group takes into account the ability of certain drugs to improve poor prognosis [14,33,76]. It is worth noting that a drug might also worsen the outcome, such as in the case of thalidomide given to groups with high-risk del(13) and del(17p) [34].

In summary, risk stratification groups are fluid and change as new treatments become available that can overcome or improve prognostic disadvantage, and new prognostically significant biomarkers are identified, leading to new categories being defined [13]. In 2014, the IMWG could not recommend any treatment decision based on risk stratification, with the possible exception of bortezomib and t(4;14) lesions. However, it can influence decisions, making potent and/or expensive treatments harder to justify in low-risk patients [37]. Naturally, the patient’s age and fitness, in particular the presence of co-morbidities and their personal priorities are of great importance [77,78]. Avet-Loiseau et al. described a low-risk group of one fifth of patients with low serum β2-microglobulin and an absence of high-risk genetic lesions, 75% of whom survived at least eight years [76]. Such individuals may benefit from treatment with entirely novel drugs targeting the tumour cells and with low toxicities for bystander cells, avoiding the risks associated with chemotherapy [38].

## 6. Minimal Residual Disease (MRD)

MRD is a term used to denote the surviving myeloma cells left in the body after therapy that may be the source of future relapse. MRD monitoring has been a successful tool in managing other haematological malignancies such as childhood acute lymphoblastic leukaemia (ALL) and chronic myeloid leukaemia (CML), facilitating earlier treatment interventions by improved anticipation of relapse or, if absent, reduced toxicity associated with reduction in therapeutic requirements [79]. Decisions on treatment intensity in ALL use MRD [80], where, for example, the absence or presence of MRD following induction chemotherapy is linked to EFS and relapse, respectively [81]. It offers the potential benefits of risk stratification and an earlier assessment of treatment response and drug resistance [4]. Detection of MRD by flow cytometry is possible down to a level of one malignant cell against a background of 10^−5^ or even 10^−6^ normal haematopoietic cells [71], with similar, or even greater sensitivity achievable using molecular methods.

### 6.1. MRD Detection by Flow Cytometry

MRD assessment by multiparameter flow cytometry has been shown to be a powerful marker of OS in PCM, with evidence of a relationship between the quantity of MRD (number of cells) and OS [72]. In addition, time to progression (TTP) in PCM correlates with MRD level: <10^−5^ 80 months TTP; 10^−3^–10^−5^ 48 months TTP; >10^−3^ 27 months TTP [71]. MRD assessment therefore yields important information of prognostic value, namely depth of response and number of residual tumour cells [38,82]. It provides a measure of tumour heterogeneity over time by monitoring immunophenotypic shifts [4]. Two studies have shown 14.5% and 36% of patients showing an apparent complete response have MRD [74,83]. In multivariate analyses, only MRD and cytogenetics (iFISH) are independent markers for PFS and OS [72].

### 6.2. MRD Detection Using Molecular Methods

Molecular methods can be used as an alternative to flow cytometry for the detection of minimal residual disease. The ASO PCR method can detect myeloma cells at a sensitivity of one cell in a million, currently beyond the capability of most flow cytometry protocols [35].

Drugs are usually used in combinations and identifying the most effective for each subtype and stage of disease continues to be the subject of clinical trials. As discussed above, PCM is known to have a higher genetic heterogeneity compared to other haematological malignancies. Thus, a plasma cell tumour is likely to contain subclones with different levels of drug resistance. Variation increases with progression and with increased risk [57]. Although no link has been found to date between clonal heterogeneity and OS, there is a link between the overall number of mutations and a reduced PFS and OS [60]. A cocktail of anti-cancer agents might therefore have greater efficacy and indeed the level of tumour heterogeneity might in future be used to decide the number of different agents in a treatment combination [30,31].

### 6.3. Characterisation of MRD

There is a strong likelihood that MRD cells are drug resistant [55]. MRD cells may be “silent”, i.e., not secreting paraprotein and so impossible to detect via clinical biochemical means [55]. These cells may also have unique cytogenetic, genetic and immunophenotypic profiles [55]. There is immunophenotypic evidence suggesting they may be a subset of the cell population seen at diagnosis, with high expression of pro-survival and adhesion molecules, CD138, CD49e, CD44, CD54, CD29, CD11a, CD11c, CD49d, CXCR4, and HLA-DR [55].

Flow cytometry also offers a highly sensitive means of detecting circulating malignant plasma cells in PB. The presence of even low numbers of such cells is an indicator of poor prognosis in newly diagnosed, treated and relapsing PCM patients. Their detection in large numbers is also a key component of the diagnosis of PCL, although this is usually diagnosed initially by morphological examination of a PB smear stained using a Romanowsky staining procedure [42,84,85].

#### 6.3.1. Surface Markers with Known Prognostic Significance

The expression of many individual surface proteins has known prognostic significance, demonstrating a correlation with survival or disease progression. CD27 is expressed on normal plasma cells although less commonly in PCM and, when present, expression is often heterogeneous and lower at relapse [86]. CD117 is expressed on clonal plasma cells in most cases of MGUS, on fewer cases of SMM and fewer still of PCM [87,88]. Expression of any of CD19, CD81, CD82 or CD86 has been reported to be an indicator of poor prognosis [89,90]. CD28 expression is associated with the cytogenetic aberrations t(14;16) and del(17p) [91], while absence of CD117 expression is associated with t(4;14) and del(13q) [91]. Furthermore, using IHC on BM trephine biopsy samples, CD99 expression has been shown to correlate with longer OS [92], whilst IHC has been used to assess Erk activation relevant to Ras signalling, which is of use to inform decisions on the use of B-Raf inhibitors [29].

CD56, CD44, CD11a, CD49e, CD45RO, and CD45RA are adhesion molecules and, in malignancy, may be involved in spread of the tumour, immortalisation of the tumour cells and also expressed on normal plasma cells [93]. CD56 is overexpressed in PCM but reduces in extramedullary spread [93]. CD11a, CD49e, CD45RA and CD45RO may be expressed on a sub-population of tumour cells, characterised by lower malignancy and is further reduced during disease progression [93]. In PCM, CD49d shows bright uniform expression, whilst expressions of CD44 and CD184 are more heterogeneous [94].

Paino et al. (2015) examined 116 PCD patients over different disease time-points using a 23-colour flow cytometry method and found 35 of them had subclonal cell populations, of which 10 had MRD, with five showing “clonal tiding” after therapy, i.e., alteration in the proportions of different subclones [95]. This has also been seen using molecular techniques.

#### 6.3.2. Altered Gene Expression in MRD or at Relapse

A recent genomic study on MRD with bortezomib resistant cells showed an overall deregulation of gene expression and specifically reduced expression of genes involved in the processing of protein such as proteasome subunits and the endoplasmic reticulum [55]. *FNLA* and *FERMT3* upregulation and *ALCAM* downregulation in MRD compared to PCM at diagnosis has been linked to reduced OS [55] whilst *NFκB* has been reported to be upregulated at relapse [28].

When secondary genetic changes are identified at relapse these may inform choice of specific therapy. For example, when newly acquired del(17p) is identified, pomalidomide has shown efficacy. When a newly acquired t(4;14) is present, the use of a proteasome inhibitor is indicated.

## 7. Future Developments

Cell surface antigens on myeloma cells offer tempting drug targets as mAbs can be used. Target surface proteins need to be as exclusive to the myeloma cells as possible to reduce toxicity to other haematopoietic and non-haematopoietic cells, and ideally have universal, consistent and homogeneous expression on the target cells. The protein must also form an important function for the tumour cell to prevent it being downregulated to evade drug induced death without negative consequences for the cell.

Flow cytometric immunophenotyping can be used to identify which potential targets the individual tumour possesses and in this way risk stratification could be greatly expanded using a panel of mAbs to a wider range of antigens, with the presence of the set of targets associated with a level of risk and each of the corresponding antibody treatments reducing that risk. The continued expression of the target throughout treatment also would need to be confirmed, necessitating repeat immunophenotyping and alternative myeloma signature surface markers would be required where important original markers such as CD38 and CD138 are targeted.

There are several antigens with mAbs available relevant to plasma cell myeloma but currently only the antibodies to CD38, daratumumab and SAR650984, have proved their efficacy as myeloma treatment options [48]. These were tested as monotherapy on relapsed/refractory myeloma. Antibodies to other antigens such as CD319 (elotuzumab) and CD138 (BT-062) have shown less impressive results but may still be effective as part of combination therapy for treating MGUS or earlier stage PCM [48,96]. Other markers with potential as mAb targets include CD74 (milatuzumab), CD54, CXCR4, B cell-activating factor (BAFF) and interleukin-6 (IL-6) [58,96]. Although elotuzumab as monotherapy in PCM did not show particularly impressive efficacy, in phase II and phase III trials in combinations it was shown to be more effective in less treated PCM patients and is undergoing study as monotherapy for SMM [48]. IL-6 is a pro-inflammatory cytokine involved in survival and proliferation in PCM and a potential target for mAb therapy but results to date suggest it may be more important early on in the disease [48].

Chimeric antigen receptor (CAR) T cell therapy has been successful in other malignancies leading to interest in development for use in myeloma. For mAb therapy, immunophenotypic data provided by flow cytometry will be of great value in determining appropriate antigen targets in individual patients [96]. As the CAR T cell specificity will be based on mAbs currently being developed and trialled as therapies in their own right, their efficacy and toxicity will be of great relevance to decisions about their potential use in CAR T cell therapies. There are several potential surface markers that could form part of the T cell target. The anti CD19 mAb already forms part of successful CAR T cell therapy in B cell lymphoma and may be effective against late stage CD19 expressing PCM [96]. CD269 may be a good CAR T target due to its relatively restricted expression on plasma cells and its importance for proliferation and survival [5]. Other markers such as CD138, CD56 and CD314 have potential, albeit with suboptimal specificity for myeloma cells [5]. Evidence of toxicity of CAR T treatments has led to a delay in their development so that suicide genes can be incorporated as a safety feature, while expression of target markers on the therapeutic T cells is also a problem [5].

Targeting mutations may be flawed if heterogeneity means selection for a subclone occurs. Furthermore, more than one mutation in the same pathway could likewise render targeting only one of them useless [26]. However, development work, and clinical trials continue, including studies using signal transduction pathway inhibitors [58], and the PI3K/AKT/mTOR, MAPK, JAK-STAT, and NFκB pathways, which are activated in PCM, are under investigation. The NFκB pathway is targeted already by proteasome inhibitors. AKT inhibitors under study include perifosine and GSK211083; everolimus and temsirolimus target mTOR; tipifarnib inhibits RAF activation; selumetinib inhibits MEK; SCIO-469 inhibits MAPK; plitidepsin inhibits c-Jun. It is estimated that 4% of PCM patients have an activating mutation in *BRAF* and could be a candidate for treatment with B-Raf inhibitors.

In addition to study of lncRNAs and miRNAs in furthering understanding of the biology of PCD, diagnostics and risk stratification, as described above, miRNA therapeutics is showing great promise in the search for more stratified, or personalised treatments in PCM [17,19,20]. This has resulted in the concept of theragnostics, where information obtained through recent advances in laboratory based methodologies, and discoveries resulting from them, informs and is combined with novel therapeutics to optimise patient outcome, with the least possible toxicity [17]. This approach utilises synthetic miRNA mimics which can be used in the first instance to restore the normal functional levels of those miRNAs such as miR-192 and miR-34a, which are downregulated in PCM, or to antagonise those which are overexpressed [17,19]. Furthermore, recent studies have suggested the use of miRNAs that can restore or inhibit the classically altered genetic pathways in PCM, such as *IGH*, *FGFR3*, *MMSET*, and actually reprogram the malignant cells [19,20]. A less disease specific but wholly effective approach has also been proposed which targets more general pathways of tumour growth and cell death that may not be specific to PCM, for example by using miR-199-5p to inhibit tumour growth [18,20]. A hallmark of miRNAs is that each one can usually target several different genes in a coordinated approach, affecting entire pathways and cell function networks [20]. Hence the use of miRNAs, rather than other genetic based therapies such as small interfering RNAs (siRNAs), promises to have a more significant effect on the tumour cell biology by targeting the same pathways and features on multiple genetic fronts [19,20].

Protein biomarkers have been sought using mass spectrometry on blood samples from myeloma patients and healthy controls to identify differentially expressed proteins. Dowling et al. focused on biomarkers of bone disease in myeloma and identified 24 proteins (six of which were downregulated) including complement proteins and extracellular matrix glycoproteins [97], while Zhang et al. found 22 proteins (10 downregulated) [98].

Proteomics studies have also identified treatment response pathways that may be useful to predict treatment response. There is considerable variability between proteomics platforms as well as incomplete proteome coverage. Consensus between the different platforms can be used to identify proteins whose expression is different in those who respond well to treatment compared to those who do not. This approach has identified pathways involved in ER stress response, mitochondrial function and acute phase response involving IL-6, but there is a need for validation and little correlation with GEP [99]. miRNAs may be able to help resolve these differences since these interactions have also been shown to affect cellular pathways at a post transcriptional level [16,20]. miRNA based therapies could also hold the key to providing the pathway level cellular perturbations necessary for truly effective personalised treatment which takes these genomic-proteomic differences into account [18,19,20].

## 8. Summary

Advances at the bench have made significant contributions to the care of patients diagnosed with PCD. These include improved understanding of the biology of the disorders, and in their diagnosis, sub-classification, risk stratification, monitoring of treatment, and detection of MRD and early relapse. Although PCM is still considered to be incurable using treatments currently available, these contributions have been a vital component in the progress made to date towards improved survivals. The expectation is that this progress will continue, with the realistic possibility of redefining the disorders as curable, at least for some subgroups of patients. Particularly important have been the advances in cellular and molecular biology made possible by newer methodologies such as multicolour flow cytometry and cell sorting with access to an ever-increasing range of very specific mAbs and a wider choice of fluorochromes, iFISH, PCR-based molecular techniques, and the current rapidly expanding molecular and gene expression capabilities, including NGS. This has led to an ever-increasing understanding of pathways impacted by altered expression or mutation of specific genes, the role of miRNAs and lncRNAs, and the ensuing proliferation and/or survival advantages. The contribution of the pharmaceutical industry through development of new drugs targeting these aberrations is central to the whole effort.

Although treatment decisions based on diagnostic subdivision and risk stratification remain confined mainly to the larger, research orientated clinical units or tertiary referral centres, this approach will become more widespread in the foreseeable future, particularly as surrogate markers are identified for the more complex, and expensive to perform, procedures, and new treatments are validated in clinical trials. In the past decade, the outlook for a newly diagnosed patient has improved significantly, with subgroups of patients now having an expectation of survival that will result in their eventual demise for reasons other than their PCD. However, considerable challenges remain, particularly in the context of the substantial minority of patients whose survival remains disappointingly short. Furthermore, some of the novel treatments now in use have considerable toxicities and reduced toxicity with increased efficacy remains the goal.

## Figures and Tables

**Table 1:** International Myeloma Working Group 2014 Definition of SMM and PCM [7].

Plasma Cell Myeloma
One or both of
≥10% clonal plasma cells in the BM
Extramedullary plasmacytoma
One or more of the following CRAB criteria and biomarkers of malignancy
Hypercalcaemia (C) as defined by serum calcium >0.25 mM above the normal range or >2.75 mM
Renal insufficiency (R) as defined by creatinine clearance <40 mL/min or serum creatinine >177 µM
Anaemia (A) as defined by haemoglobin >20 g/L below the normal range or <100 g/L
≥1 bone lesion (B) as detected by radiography, computed tomography (CT) or Positron Emission Tomography-Computed Tomography (PET-CT)
Bone marrow consisting of ≥60% clonal plasma cells as calculated by ĸ/λ light chain restriction flow cytometry, immunohistochemistry (IHC) or immunofluorescence (IF)
Involved:uninvolved serum free light chain ration ≥100
>1 focal lesion by magnetic resonance imaging (MRI)
Smouldering Multiple Myeloma
One or more of Monoclonal IgG or IgA ≥30 g/L in serum
Urinary monoclonal protein ≥500 mg/24 h in urine
Bone marrow consisting of 10%–60% clonal plasma cells as calculated by κ/λ light chain restriction flow cytometry, immunohistochemistry (IHC) or immunofluorescence (IF)
Not defined as plasma cell myeloma or amyloidosis

**Table 2:** IMWG revised International Staging System (R-ISS) [41].

Stage	Criteria	5 Year Survival
I	ISS-1 No high-risk chromosomal aberrations by iFISH Normal lactate dehydrogenase	80%
II	Neither Stage I nor III	60%
III	ISS-3 One or more high-risk chromosomal aberration by iFISH or high lactate dehydrogenase	40%
High-risk chromosomal aberrations are defined as del(17p), t(4;14), t(14;16)		

**Table 3:** mSMART risk stratification system [14].

Risk	Criteria	Median OS
High	High-risk chromosomal aberrations del(17p), t(14;16), t(14;20) or high-risk GEP	3 years
Intermediate	Chromosomal aberrations t(4;14), del 13, Hypodiploidy or PCLI >3%	4–5 years
Standard	Not the above	8–10 years
